# Mesenchymal stromal cells-derived extracellular vesicles in cartilage regeneration: potential and limitations

**DOI:** 10.1186/s13287-025-04135-6

**Published:** 2025-01-23

**Authors:** María Piñeiro-Ramil, Iván Gómez-Seoane, Ana Isabel Rodríguez-Cendal, Isaac Fuentes-Boquete, Silvia Díaz-Prado

**Affiliations:** 1 Grupo de Investigación en Terapia Celular y Medicina Regenerativa, Instituto de Investigación Biomédica de A Coruña (INIBIC), Fundación Pública Gallega de Investigación Biomédica INIBIC, Complexo Hospitalario Universitario de A Coruña (CHUAC), Servizo Galego de Saúde (SERGAS), A Coruña, 15006 Spain; 2 Grupo de Investigación en Terapia Celular y Medicina Regenerativa, Departamento de Fisioterapia, Medicina y Ciencias Biomédicas, Facultad de Ciencias de la Salud, Universidade da Coruña (UDC), A Coruña, 15006 Spain; 3 Centro Interdisciplinar de Química y Biología (CICA), Universidade da Coruña (UDC), A Coruña, 15008 Spain; 4 Centro de Investigación Biomédica en Red de Bioingeniería, Biomateriales y Nanomedicina (CIBER- BBN), Madrid, 28029 Spain

**Keywords:** OA, Exosomes, Chondrocytes, Cell-free therapy

## Abstract

**Background:**

Articular cartilage injuries can lead to pain, stiffness, and reduced mobility, and may eventually progress to osteoarthritis (OA). Despite substantial research efforts, effective therapies capable of regenerating cartilage are still lacking. Mesenchymal stromal cells (MSCs) are known for their differentiation and immunomodulatory capabilities, yet challenges such as limited survival post-injection and inconsistent therapeutic outcomes hinder their clinical application. Recent evidence suggests that the beneficial effects of MSCs are largely mediated by their secreted small extracellular vesicles (sEVs), which have been shown to promote tissue repair and reduce inflammation. MSC-derived sEVs have shown promise in mitigating cartilage degradation and chondrocyte apoptosis, positioning them as a promising alternative to MSC-based therapies for OA treatment. This review explores the potential and limitations of MSC-derived sEVs in cartilage regeneration.

**Main text:**

This systematic review was conducted following PRISMA guidelines, with a comprehensive search of the Web of Science and Scopus databases for studies published between 2019 and 2024. A total of 223 records were identified, of which 132 articles were assessed for eligibility based on general selection criteria. After full-text screening, 60 articles were initially selected, comprising 58 in vitro studies and 40 in vivo studies. Following further exclusion based on specific criteria, 33 in vitro and 28 in vivo studies from a total of 47 scientific papers were included in the final qualitative synthesis. Most studies indicate that MSC-derived sEVs enhance chondrocyte proliferation, improve cartilage extracellular matrix composition, and reduce matrix-degrading enzymes and inflammation, thereby delaying OA progression.

**Conclusion:**

A growing body of evidence supports the use of MSC-derived sEVs as a therapeutic tool for preventing OA progression, with most studies reporting beneficial effects on cartilage structure and function. However, challenges remain in optimizing and standardizing sEVs isolation, dosage, and delivery methods for clinical application. Further research is necessary to elucidate the mechanisms underlying sEVs-mediated cartilage regeneration and to facilitate their translation into effective OA therapies.

**Supplementary Information:**

The online version contains supplementary material available at 10.1186/s13287-025-04135-6.

## Background

Articular cartilage injury presents a significant challenge in orthopaedic medicine due to its limited intrinsic healing capacity. Joint lesions, whether arising from trauma or degenerative processes, can activate maladaptive repair responses and pro-inflammatory pathways, disrupting cartilage homeostasis and accelerating degeneration. Cartilage damage increases the risk of osteoarthritis (OA), which leads to pain and stiffness and severely impacts quality of life. The progressive loss of cartilage structure and function eventually results in joint space narrowing and compromised joint mobility. Despite significant research efforts aimed at discovering new therapeutic options for cartilage repair, including the transplantation of mesenchymal stromal cells (MSCs), there is currently no effective therapy that consistently regenerates articular cartilage in clinical settings.

MSCs are multipotent cells that can be isolated from adult tissues and have the ability to differentiate into a variety of cell types, including chondrocytes, osteoblasts, and adipocytes. Beyond their differentiation capacity, MSCs exhibit potent immunomodulatory and anti-inflammatory effects, making them promising candidates for cartilage regeneration. However, drawbacks such as short survival time after intra-articular injection, inconsistent therapeutic outcomes, and the risk of secondary effects, including immunological reactions, reduce the suitability of MSC transplantation [1]. Recent studies have shown that the beneficial effects of MSCs on OA joints are primarily exerted through paracrine signalling, rather than through integration into the damaged tissue. These paracrine signals are the ones that inhibit inflammatory responses and promote chondrocyte matrix regeneration [2]. In this context, MSC-derived extracellular vesicles have been shown to possess regenerative and immunomodulatory properties similar to their parent cells [3], but with lower immunogenicity and fewer safety concerns, making them an attractive alternative to traditional cell-based therapies for cartilage regeneration.

Extracellular vesicles (EVs) are lipid bilayer membrane-delimited nanoparticles secreted by all cell types, capable of influencing processes such as proliferation, differentiation, and metabolism in target cells through the transfer of bioactive molecules, including proteins and miRNAs. Based on their size, EVs are generally classified as small (< 200 nm) or large (> 200 nm) [4]. The therapeutic effects of MSC-derived EVs have been primarily attributed to small extracellular vesicles (sEVs), which are the most abundant, considered to be the active component, and are generally regarded as safe for clinical application [5]. Specifically, MSC-derived sEVs have demonstrated protective effects against cartilage degradation, making them a promising therapeutic tool in the treatment of OA and other cartilage-related disorders.

Due to their advantages over MSCs, MSC-derived sEVs are being increasingly studied as a replacement for MSCs in cartilage regenerative medicine. Additionally, unlike other common sources of sEVs, such as plasma and platelets, MSCs can be easily preconditioned or bioengineered to produce sEVs with enhanced properties. Nonetheless, their translation into clinical practice is still in its early stages [6]. During the timeframe under review, three systematic reviews exploring the potential of MSC-derived EVs for cartilage regeneration were published [7–9]. However, the most recent of these dates from 2021, while numerous new studies emerged in the following years. Furthermore, all three reviews focused solely on in vivo studies. In this review, we assess the therapeutic potential of MSC-derived sEVs for cartilage regeneration, considering both in vitro and in vivo studies, and discuss the challenges remaining in harnessing them for clinical applications.

## Materials and methods

This systematic review was elaborated following Preferred Reporting Items for Systematic Reviews and Meta-Analyses (PRISMA) guidelines [10, 11]. The search was carried out employing Web of Science and Scopus databases from 1st January 2019 to 30th April 2024. The keywords used were “extracellular vesicles” AND “mesenchymal stromal cell” AND “cartilage”. Only original research studies published as full-text articles in peer-reviewed journals were included in the analysis. Non-English publications, conference abstracts, editorials, and notes were excluded, and duplicates were removed.

The study selection criteria were as follows.

### Inclusion criteria

A. Small EVs were obtained from native, primary MSCs derived from adult tissues.
B. The method used for EVs isolation is clearly described and suitable for the isolation of sEVs, and the resulting sEVs are sufficiently characterized (at least size distribution and typical surface markers expression).
C. The article presents in vivo and/or in vitro data about the effect of MSC-derived sEVs on cartilage and/or chondrocytes.
D. The concentration or quantity (number of particles or µg of protein) of sEVs administered was specified.

### Exclusion criteria

A. Small EVs were not derived from adult MSCs, but from a different cell type or from genetically-engineered MSCs.
B. MSCs were not cultured in serum-free or EV-free medium for sEVs isolation (to avoid artefacts due to serum-derived sEVs).
C. The effect of MSC-derived sEVs was not investigated on cartilage and/or chondrocytes.
D. The effect of the whole secretome, rather than isolated sEVs, was analysed.
E. The population of isolated particles included a significant proportion (> 20%) of other type of EVs, such as large EVs (> 200 nm).
F. The method used for EVs isolation is insufficiently described or is expected to also yield large EVs due to low specificity (e.g., filter concentration alone).

For inclusion in the qualitative synthesis, specific selection criteria were applied. Regarding in vitro studies, the specific inclusion criteria were:

A. The effect of EVs was assessed directly in chondrocyte monocultures (not co-cultures including other cell types of the joint or their derived products, such as supernatant).
B. Chondrocytes used were clearly characterized in terms of their source (e.g., human, mouse, rat), phenotype (e.g., primary chondrocytes, cell lines), and health status (e.g., healthy vs. OA chondrocytes).
C. The functional outcomes are relevant to chondrocyte function (e.g., extracellular matrix (ECM) synthesis, cell proliferation and apoptosis, inflammation…) and/or sEVs interaction with cartilage (e.g. internalization by chondrocytes or penetration into the tissue).

For in vivo studies, the specific inclusion criteria were:

A. Small EVs were administered locally into the joint.
B. Small EVs were administered as a cell-free preparation.
C. Control groups (at least sham and vehicle-only groups) were included for relevant comparisons.
D. Histological evidence of cartilage degradation, where the articular surface is clearly visible, is presented for the untreated group.

Relevant data were extracted from the studies that met the inclusion criteria. For studies where numerical data were presented in graphical format but not explicitly provided in the text, PlotDigitizer software (https://plotdigitizer.sourceforge.net/) was employed to obtain numerical values. To verify that the fraction of EVs used in each study was within the sEV size range (i.e., < 200 nm), we examined the size distribution data provided in each article.

## Results

### Included studies

**Fig. 1:**
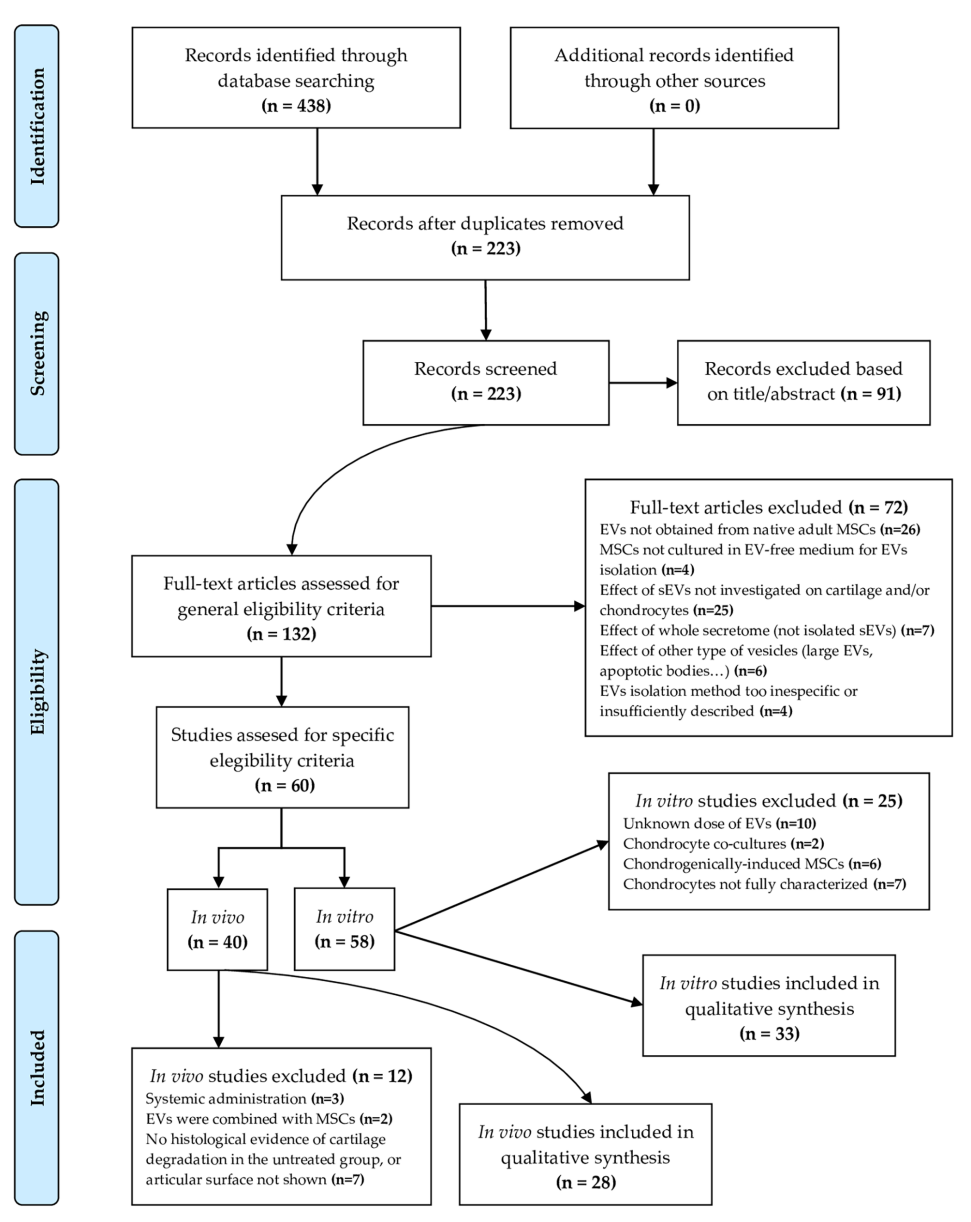
PRISMA flow diagram

A total of 223 records were retrieved from the Web of Science and Scopus databases after the removal of duplicates. Subsequently, 91 records were excluded based on their titles and abstracts, leaving 132 articles to be assessed for general eligibility criteria. After a full-text screening, 60 scientific papers that met these criteria were selected. This initial selection included 58 in vitro studies and 40 in vivo studies. Following the exclusion of studies that did not meet the corresponding specific selection criteria, 33 in vitro studies and 28 in vivo studies, from a total of 47 scientific papers, were included in the qualitative synthesis. The PRISMA flow diagram [10] is shown in Fig. 1.The basic information about the 47 selected studies is summarised in Table 1. Only two of these studies reported any conflicts of interest.

**Table 1:** Summary of selected studies characteristics

Ref.	Year	Country	Producing cells	*Cell culture conditions*				sEVs isolation method	Yield	Type of study
				Medium	Type	Duration	Stimuli			
[13]	2024	China	ADSCs (human)	MSC Medium 5% EV-free FBS	2D/3D	48 h	Gelatin methacryloyl scaffolds (3D)	Differential ultracentrifugation	2–3 µg/ml (2D) 10–15 µg/ml (3D)	In vitro
[17]	2023	Japan	ADSCs (human)	CMDs (serum-free)	2D	48 h	-	Filter concentration of filtered supernatant	5 × 10^8^ EVs/ml 50 µg/ml	In vitro, in vivo
[49]	2023	USA	ADSCs (human)	MSC Growth/ DMEM/hPL (EV-free)	2D	-	hPL	Differential ultracentrifugation	3–4 × 10^9^ EVs/ml	In vivo
[51]	2022	Canada	ADSCs (human)	CDM (PPRF-msc6) (serum-free)	2D/3D	72–84 h	Shaking (3D)	Differential ultracentrifugation	3 × 10^8^ EVs/ml 1.5 × 10^3^ EVs/cell	In vivo
[46]	2021	Italy	ADSCs (human)	α-MEM serum-free	2D	48 h	IL-1β (1 ng/ml)	Differential ultracentrifugation	-	In vitro
[24]	2021	Spain	ADSCs (human)	DMEM/F12 15% EV-free human serum	2D	48 h	-	Differential ultracentrifugation	-	In vitro
[19]	2020	Italy	ADSCs (human)	DMEM serum-free	2D	72 h	-	Differential ultracentrifugation	2.6 × 10^2^ EVs/cell 1.36 µg/10^6^ cells	In vitro
[14]	2020	Italy	ADSCs (human)	DMEM serum-free	2D	48 h	-	Differential ultracentrifugation	-	In vitro
[15]	2020	Italy	ADSCs (human)	DMEM serum-free	2D	48 h	-	Differential ultracentrifugation	-	In vitro
[31]	2020	South Korea	ADSCs (human)	DMEM serum-free	2D	24 h	-	Filter concentration of filtered supernatant	-	In vitro, in vivo
[32]	2022	China	ADSCs (mouse)	MSC Medium serum-free	2D	24 h	Curcumin (10 µM)	Differential ultracentrifugation	-	In vitro, in vivo
[43]	2024	China	ADSCs (rat)	DMEM/F12 10% EV-free FBS	2D	48 h	-	Differential ultracentrifugation	-	In vitro, in vivo
[34]	2021	China	ADSCs (rat)	α-MEM 10% EV-free FBS	2D	48 h	-	Density gradient ultracentrifugation	-	In vitro
[52]	2023	The Netherlands	BMSCs (human)	α-MEM 5% EV-free hPL	2D	48 h	-	Differential ultracentrifugation	38.8 EVs/cell	In vivo
[30]	2022	China	BMSCs (human)	DMEM serum-free	2D	-	-	Differential ultracentrifugation	-	In vitro
[44]	2022	China	BMSCs (human)	α-MEM serum-free	2D	48 h	-	Exosome Purification Kit	-	In vitro, in vivo
[27]	2022	China	BMSCs (human)	DMEM/F12 10% EV-free FBS	2D	48 h	Hypoxia	Differential ultracentrifugation	-	In vitro, in vivo
[22]	2021	Germany	BMSCs (human)	α-MEM 10% EV-free FBS	2D	48 h	-	Differential ultracentrifugation	3–4 × 10^10^ EVs/ml	In vitro
[66]	2021	China	BMSCs (human)	DMEM 10% EV-free FBS	2D	48 h	-	Exosome Purification Kit	-	In vitro
[38]	2021	China	BMSCs (human)	CMD (UR51101) (serum-free)	2D	48 h	-	Exosome Purification Kit	-	In vitro, in vivo
[23]	2021	United Kingdom	BMSCs (human)	DMEM 5% EV-free hPL	2D	48 h	-	Differential ultracentrifugation	1.38 × 10^11^ EVs/ml 635 × EVs/cell (OA) 1.12 × 10^11^ EVs/ml 709 EVs/cell (healthy)	In vitro
[33]	2021	China	BMSCs (human)	DMEM serum-free	2D	48 h	-	Filter concentration of filtered supernatant	1.05 × 10^7^ EVs/ml	In vitro
[21]	2020	Germany	BMSCs (human)	α-MEM 10% EV-free FBS	2D	48 h	-	Differential ultracentrifugation	-	In vitro
[12]	2020	Italy	BMSCs (human)	α-MEM serum-free	2D	72 h	FBS/XFS	Differential ultracentrifugation	0.5 µg/10^6^ cells 15 EVs/cell (FBS) 1.5 µg/10^6^ cell 30 EVs/cell (XFS)	In vitro
[54]	2024	China	BMSCs (mouse)	DMEM/F12 10% EV-free FBS	2D	-	EMF/USPIO	Differential ultracentrifugation	-	In vivo
[40]	2022	China	BMSCs (mouse)	DMEM serum-free	2D	48 h	-	Differential ultracentrifugation	-	In vitro
[47]	2023	Iran	BMSCs (rabbit)	DMEM 10% EV-free FBS	2D/3D	-	Chondrogenesis Chondrocyte co-culture	Differential ultracentrifugation	21.23 µg/10^6^ cells (2D) 0.83 µg/10^6^ cells (3D)	In vivo
[59]	2022	Iran	BMSCs (rabbit)	DMEM 10% EV-free FBS	3D	7 days	Shaking	Size-exclusion chromatography	10 µg/10^6^ cells	In vivo
[29]	2024	China	BMSCs (rat)	DMEM/F12 10% EV-free FBS	3D	-	SA/HA Hydrogels	Ultracentrifugation of filtered supernatant	300 EVs/cell (2D) 1.5 × 10^3^ EVs/cell (3D)	In vivo
[26]	2021	China	BMSCs (rat)	DMEM/F12 10% EV-free FBS	2D	48 h	Hypoxia	Filter concentration of filtered supernatant	-	In vitro, in vivo
[67]	2021	Italy	BMSCs vs. ADSCs (human)	DMEM/α-MEM serum-free	2D	24 h	-	Differential ultracentrifugation	-	In vitro
[39]	2022	Italy	BMSCs vs. SFMSCs (horse)	DMEM 10% EV-free FBS	2D	24 h	-	Ultracentrifugation of filtered supernatant	1.9 × 10^10^ EVs/ml (BMSCs) 1.7 × 10^11^ EVs/ml (SFMSCs)	In vitro
[36]	2023	China	DFCs (rat)	MSC Medium serum-free	2D	48 h	-	Exosome Purification Kit and filter concentration	-	In vitro
[50]	2023	USA	SFMSCs (rat)	DMEM serum-free	2D	48 h	-	Differential ultracentrifugation	7.5 × 10^7^ EVs/ml	In vivo
[25]	2021	China	SMSCs (human)	DMEM serum-free	2D	48 h	LPS (100 ng/ml)	Differential ultracentrifugation	-	In vitro, in vivo
[41]	2021	China	SMSCs (human)	DMEM 10% EV-free FBS	2D	48 h	-	Differential ultracentrifugation	-	In vitro, in vivo
[53]	2021	China	SMSCs (human)	CDM (StemGro) (serum-free)	2D	48 h	-	Density gradient ultracentrifugation	-	In vivo
[16]	2023	Spain	UCMSCs (human)	DMEM serum-free	2D	48 h	-	Size-exclusion chromatography	2.7 × 10^10^ EVs/ml 35 µg/ml	In vitro
[35]	2023	China	UCMSCs (human)	α-MEM 10% EV-free FBS	2D	48 h	-	Differential ultracentrifugation	2.2 × 10^10^ EVs/ml	In vitro, in vivo
[55]	2023	China	UCMSCs (human)	StemXVivo 10% EV-free FBS	2D	48 h	-	Differential ultracentrifugation	0.5 µg/ml	In vivo
[56]	2022	China	UCMSCs (human)	MSCYF01-500/ MSCYF02-20	2D	-	-	Size-exclusion chromatography	-	In vivo
[57]	2022	China	UCMSCs (human)	DMEM serum-free	2D	48 h	-	Differential ultracentrifugation	-	In vivo
[28]	2022	Vietnam	UCMSCs (human)	DMEM/F12 10% EV-free FBS	2D	48 h	TGF-β (10 ng/ml) TNF-α (20 ng/ml) INF-α (20 ng/ml)	Differential ultracentrifugation	-	In vitro
[58]	2022	China	UCMSCs (human)	DMEM 10% EV-free FBS	2D	48 h	-	Differential ultracentrifugation	-	In vivo
[48]	2021	China	UCMSCs (human)	α-MEM 10% EV-free FBS	2D	48 h	-	Differential ultracentrifugation	-	In vivo
[42]	2021	China	UCMSCs (human)	MSCYF01-500/ MSCYF02-20	2D	48 h	-	Size-exclusion chromatography	2.3 × 10^7^ EVs/ml (20.920 µg/ml) 61.1 EVs/cell (62.8 µg/10^6^ cells)	In vitro, in vivo
[37]	2020	China	UCMSCs (human)	12–725 F 10% EV-free FBS	2D	72 h	-	Differential ultracentrifugation	8.2 × 10^6^ EVs/ml	In vitro, in vivo

FBS: foetal bovine serum; CMD: chemically-defined medium; hPL: human platelet lysate; XFS: xeno-free supplement; EMF: electromagnetic field; USPIO: ultrasmall superparamagnetic iron oxide; SA/HA: sodium alginate/hyaluronic acid

**Fig. 2:**
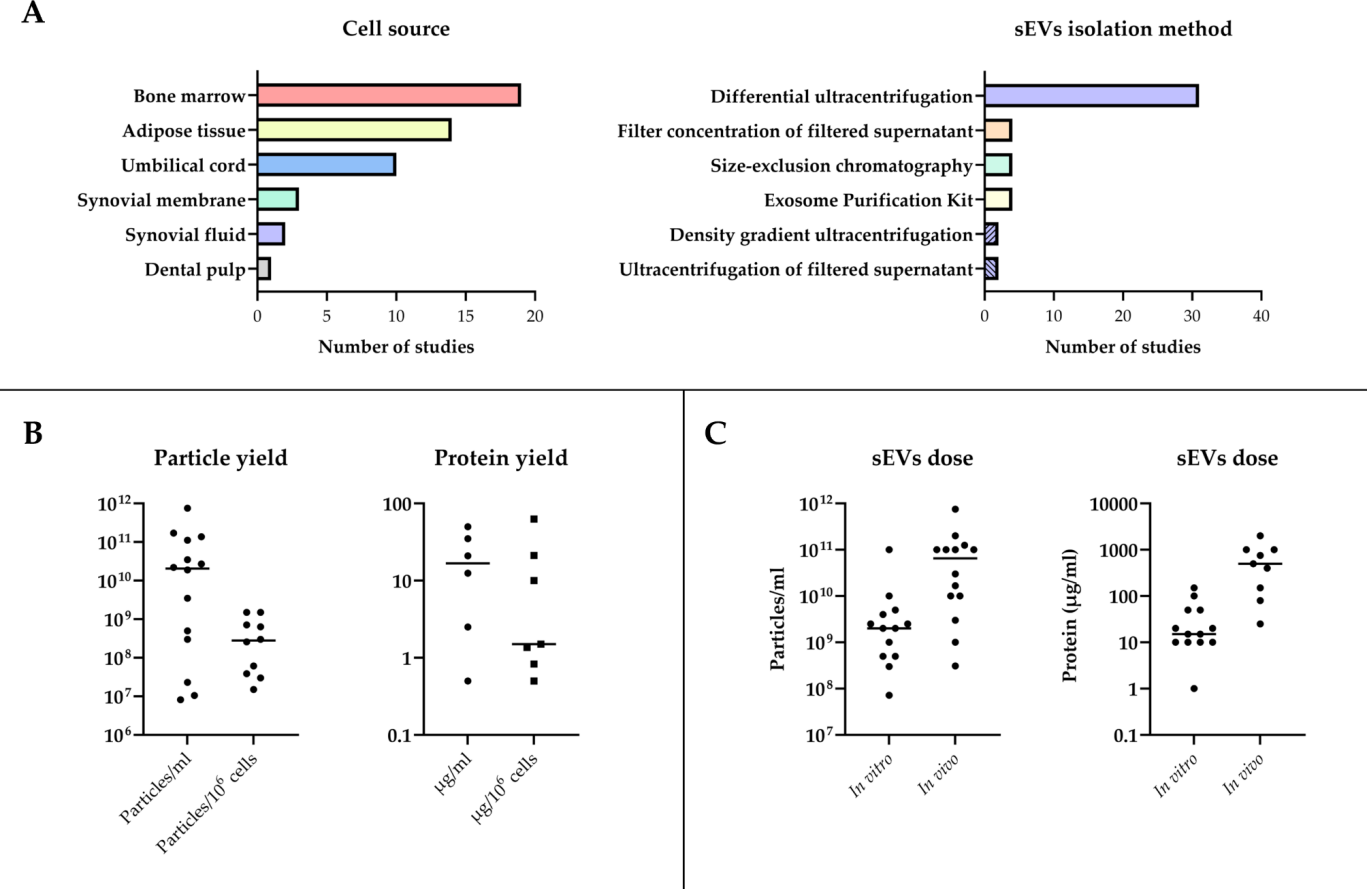
**(A)** Histograms showing the preferred cell sources and sEVs isolation methods employed in the selected studies. **(B)** sEVs yield, in terms of both number of particles and protein quantity. **(C)** sEVs dose employed in the in vitro and in vivo studies, in terms of both number of particles and protein quantity

A total of 26 papers originated from China, making it the most represented country in this review. Italy followed with 7 papers, while several other countries were represented by one or two papers. Among the selected studies, bone marrow MSCs (BMSCs) were the preferred sEVs source (19 studies), followed by adipose tissue-derived MSCs (ADSCs, 14 studies), umbilical cord MSCs (UCMSCs, 10 studies), synovium-derived MSCs (SMSCs, 3 studies), synovial fluid MSCs (SFMSCs, 2 studies), and dental follicle cells (DSCs, 1 study). The preferred isolation method was differential ultracentrifugation, used in 31 studies (Fig. 2A). Reported yields vary, ranging from 0.5 to 50 µg/mL and 10^7^-10^11^ particles/mL, with filter concentration of filtered supernatant generally leading to higher yields in terms of concentration of protein, but not necessarily in terms of pure sEVs. In terms of yield per million cells, reported quantities varied from 10^7^ to 10^9^ particles and from 0.5 to 60 µg (Fig. 2B). Some authors have shown that sEVs yield depends on culture conditions [12, 13]. Only five studies specifically reported purity [12, 14–17] (number of particles per µg of protein) [18], and one of them noted that it may vary up to two orders of magnitude depending on the culture medium used [17].

### Effects on chondrocytes and Cartilage: in Vitro evidence

The effect of MSC-derived sEVs on cartilage cells has been extensively investigated in various in vitro models. This systematic review includes 34 in vitro studies (Table 2), most of which employed primary human articular chondrocytes from OA patients as target cells (12 studies). Of the 7 studies which specified the sex of the cartilage donors [14, 19–24], 5 reported employing a majority of females [14, 21–24]. Additional studies involved articular chondrocytes from rats (6 studies), mice (4 studies), rabbits, and horses (1 study each). Several studies were performed ex vivo on human articular cartilage explants (2 studies) and mouse foetal growth cartilage (1 study). Other cell sources included nucleus pulposus cells (NPCs) from intervertebral disc degeneration (IVDD) patients, costal chondrocytes from paediatric patients, and mandibular condylar chondrocytes from rats and rabbits (1 study each). Additionally, 3 studies used human chondrocyte cell lines. The sEVs employed in these studies were mostly derived from human BMSCs (11 studies), ADSCs (9 studies), UCMSCs (5 studies), and SMSCs (3 studies). Additional cell sources included rodent BMSCs (2 studies) and ADSCs (3 studies), horse BMSCs and SFMSCs, and rat DSCs (1 study each).

**Table 2:** Summary of in vitro studies characteristics and outcomes

Ref.	Producing cells	In vitro model	Results
[16]	UCMSCs (human)	Articular cartilage (human, healthy)	**10**^**9**^**EVs/explant** (14 days) No changes in ECM content (proteoglycans/collagen)
[46]	ADSCs (human)	Articular cartilage (human, OA, 38–54 y/o)	**2.5 × 10**^**9**^**EVs/ml** (40 h) sEVs preconditioned with IL-1β have chondro-protective miRNA content EVs penetrate in micromasses and cartilage explants (40 μm depth)
[39]	BMSCs vs. SFMSCs (horse)	Articular chondrocytes (horse, healthy, 5–10 y/o)	IL-1β/TNF-α + **4 × 10**^**9**^**EVs** (24 h) Reduced MMP13 [qPCR] (BMSC-EVs only)
[17]	ADSCs (human)	Articular chondrocytes (human, healthy)	**5 × 10**^**9**^**EVs/mL** (3 days) Increased cell proliferation and migration and reduced apoptosis; Increased COL1, COL2 [qPCR]
[25]	SMSCs (human)	Articular chondrocytes (human, OA)	IL-1β + **10**^**10**^**EVs/ml** (24–48 h) Increased cell proliferation and migration and reduced apoptosis Increased ACAN, COL2A1; reduced ADAMTS5 [Western blot]
[28]	UCMSCs (human)	Articular chondrocytes (human, OA)	**10 µg/ml** (2–7 days) No effect on cell proliferation (48 h) No change in COL2; increased ACAN, RUNX2 [qPCR] (1 week)
[12]	BMSCs (human)	Articular chondrocytes (human, OA)	IL-1α + **1 µg/ml EVs** (16–48 h) Internalization efficiency increases with IL-1α Reduced IL8, IL8, COX2 at 16 h but no effect at 48 h
[31]^†^	ADSCs (human)	Articular chondrocytes (human, OA)	IL-1β + **10**^**8**^**EVs** (24–72 h) Increased cell proliferation and migration Reduced MMPs, ADAMTS5; increased COL2 [qPCR] Reduced MMPs [ELISA]
[14]	ADSCs (human)	Articular chondrocytes (human, OA, 38–54 y/o)	Chondrogenesis + **2.5 × 10**^**9**^**EVs/ml** (48 h) EVs fully penetrate chondrocyte micromasses EVs quickly penetrate the surface of cartilage explants
[23]	BMSCs (human)	Articular chondrocytes (human, OA, 55–83 y/o)	**EVs from 6 × 10**^**4**^**MSCs** in 300 µl Increased cell viability **EVs from 6 × 10**^**6**^**MSCs** for 3 pellets Increased SOX9, COL2, ACAN [qPCR] (21 days)
[19]	ADSCs (human)	Articular chondrocytes (human, OA, 64 ± 13 y/o)	TNF-α + **EVs from 10**^**5**^**ADSCs** (3–6 days) Increased cell proliferation No changes in TNFα-induced MMP activity but reduced COL10 [Western blot]
[22]	BMSCs (human)	Articular chondrocytes (human, OA, 66 ± 7 y/o)	IL-1β + **10 µg/ml EVs** (24 h) Increased cell proliferation and migration and reduced apoptosis Increased COL2, SOX9, ACAN; reduced COLX, MMP13, IL6 [qPCR]
[15]	ADSCs (human)	Articular chondrocytes (human, OA, 67 ± 5 y/o)	**50**,**000 EVs/chondrocyte** ECM plays a crucial role in EV-cell interactions Outcomes can vary significantly between 2D and 3D environments
[21]	BMSCs (human)	Articular chondrocytes (human, OA, 67 ± 8 y/o)	IL-1β + **10 µg/ml EVs** (24 h) Increased cell proliferation and migration and reduced apoptosis Increased COL2, SOX9, COMP; reduced COLX, MMP13, ALPL, IL1β [qPCR]
[24]	ADSCs (human)	Articular chondrocytes (human, OA, 68 ± 8 y/o)	IL-1β + **7.2 × 10**^**7**^**EVs/mL** (24 h) Reduced oxidative stress
[32]	ADSCs (mouse)	Articular chondrocytes (mouse, healthy, 4 weeks old)	TBHP + **10**^**9**^**EVs** (24–48 h) Increased cell proliferation Reduced apoptosis and oxidative stress Increased ACAN, COL2; reduced MMP13, ADAMTS5, IL-1β, TNF-α [qPCR]
[40]	BMSCs (mouse)	Articular chondrocytes (mouse, healthy, 8 weeks old)	IL-1β + **100 µg/mL EVs** (24 h) Increased cell proliferation and migration and reduced apoptosis Reduced TNF-α, IL-6 [ELISA]
[37]	UCMSCs (human)	Articular chondrocytes (mouse, healthy, newborn)	Chondrogenesis + **10**^**9**^**EVs/mL** (21 days) Increased area of micromasses Increased ACAN, COL2 [Western blot]
[66]	BMSCs (human)	Articular chondrocytes (mouse, OA)	**200 µg EVs** Increased cell proliferation and migration and reduced apoptosis
[44]	BMSCs (human)	Articular chondrocytes (rabbit, healthy)	**5 × 10**^**8**^**− 2 × 10**^**9**^**EVs/ml** (24–48 h) Increased cell proliferation and migration Increased mitochondrial function
[26]	BMSCs (rat)	Articular chondrocytes (rat, healthy)	**150 µg/ml EVs** (24 h) Hypoxia enhances the release of sEVs from BMSCs and their uptake by chondrocytes Increased cell proliferation and migration and reduced apoptosis Increased COL2; reduced MMP13 [Western blot]
[42]	UCMSCs (human)	Articular chondrocytes (rat, healthy)	IL-1β + **15 µg/ml EVs** (24–72 h) Increased cell proliferation and migration
[43]	ADSCs (rat)	Articular chondrocytes (rat, healthy)	**2 × 10**^**9**^**EVs/ml (20 µg/ml)** (24–72 h) Increased cell proliferation and migration and reduced apoptosis
[27]	BMSCs (human)	Articular chondrocytes (rat, healthy, 4 weeks old)	IL-1β + **20 µg EVs** (24–48 h) Increased cell proliferation and migration and reduced apoptosis
[35]	UCMSCs (human)	Articular chondrocytes (rat, healthy, 5 days old)	IL-1β + **5 × 10**^**8**^**EVs/ml** (72 h) Increased ACAN, COL2 [immunofluorescence]; Reduced MMP13 [Western blot]
[43]	ADSCs (rat)	Articular chondrocytes (rat, healthy, 6–8 weeks old)	IL-1β + **50 µg/mL EVs** (48–72 h) Increased cell proliferation and migration and reduced apoptosis Increased COL2, COL1, RUNX2, osteocalcin; reduced p53, IL-1β [qPCR/Western blot]
[38]	BMSCs (human)	Cell line C28/I2 (human costal cartilage, SV40LT)	IL-1β + **10 µg/ml EVs** (24 h) Increased cell proliferation and migration and reduced apoptosis Reduced IL6; no changes in MMP13 [Western blot]
[41]	SMSCs (human)	Cell line SW1353 (human chondrosarcoma)	IL-1β + **10**^**11**^**EVs/ml** (24 + **6** hours) Increased cell proliferation and reduced apoptosis (48 h) Reduced TNF-α; increased IL-10 [ELISA]
[13]	ADSCs (human)	Costal chondrocytes (human, healthy, pediatric patients)	**15 µg/mL sEVs** (72 h) Increased cell proliferation (2D) Increased COL1, COL2 (2D and 3D) [Western blot]
[67]	BMSCs vs. ADSCs (human)	Growth cartilage (mouse, foetal)	**0.5 µg/explant** (7 days) **ADSC-EVs** promote angiogenesis **BMSC-EVs** promote chondrogenesis and growth plate organization
[33]	BMSCs (human)	Mandibular condylar chondrocytes (rabbit, healthy)	IL-1 + **2–4 × 10**^**8**^**EVs** (24 h) Increased cell proliferation and migration Increased SOX9, COL2 [qPCR/Western blot]
[36]	DFCs (rat)	Mandibular condylar chondrocytes (rat, healthy, 3–5 days old)	IL-1β + **50 µg/ml EVs** (24–96 h) Increased cell proliferation and migration and reduced apoptosis Increased ACAN; reduced MMP13 [Western blot]
[30]	BMSCs (human/rat)	Nucleus pulposus cells (human, IVDD, 25–43 y/o)	TNF-α + **100 µg/mL EVs** (48 h) Increased cell proliferation; reduced senescence and apoptosis Increased COL2, ACAN; reduced MMP13, ADAMTS5 [Western blot]

^†^: Competing interests declared by the authors

### Chondrocyte viability, proliferation, and apoptosis

Numerous studies have found that MSC-derived sEVs enhanced cell viability, proliferation and migration while reducing apoptosis in both interleukin-1 β (IL-1β)-induced and unstimulated chondrocytes, in a wide range of concentrations (Fig. 2C). Interestingly, sEVs derived from MSCs preconditioned with lipopolysaccharide (LPS) [25] or hypoxia [26, 27] demonstrated superior protective effects. In contrast, one study reported no significant effect on chondrocyte proliferation when using sEVs derived from either unstimulated or tumour growth factor-β (TGF-β)-, tumour necrosis factor-α (TNF-α)-, or interferon-α (IFN-α)-stimulated MSCs. This outcome was attributed to the low dose of sEVs employed [28], although other authors have observed positive effects on cell proliferation using the same dose of sEVs (10 µg/mL) and target cell type (human OA chondrocytes) [29]. This discrepancy may be due differences in the purity of the sEV preparations, which were not reported in either study, and/or the different cell sources used (UMSCs [28] vs. BMSCs [29]). A total of 15 studies reported that sEVs reduced chondrocyte apoptosis induced by inflammatory *stimuli*, while 2 studies demonstrated that sEV treatment also alleviated oxidative stress. Additionally, Hao et al. (2022) reported a reduction in TNF-α-induced senescence in sEVs-treated NPCs [30]. Sanjurjo-Rodríguez et al. (2021) reported a moderate increase in cell viability induced by BMSC-derived sEVs, regardless of the health status of the producing cells (healthy vs. OA) [23].

### Extracellular matrix synthesis and degradation

Regarding their effect on the anabolism/catabolism balance, sEVs have been reported to increase the expression of hyaline cartilage components such as aggrecan (ACAN) and type II collagen (COL2) at both the gene [17, 21, 31–34] and protein [25, 26, 30, 33–36] levels, while reducing the expression and secretion of matrix-degrading enzymes, including matrix metalloproteinases (MMPs) and ADAMTS5 [21, 25, 26, 30–32, 36]. MSC-derived EVs have also been reported to increase ACAN and COL2 expression after 21 days of pellet culture [23, 37], as well as the area of micromasses [37]. Sanjurjo-Rodríguez et al. (2021) noted that the increase in anabolic gene expression was more pronounced when employing healthy MSC-derived EVs compared to OA MSC-derived EVs [23]. In contrast to these results, some authors found no effect on the expression [23, 38] or activity [19] of matrix-degrading enzymes following sEV treatment. On a related note, Arévalo-Turrubiarte et al. (2021) reported that while BMSC-derived sEVs reduced MMP13 expression, SFMSC-derived sEVs did not [39]. Furthermore, sEVs derived from BMSCs cultured under hypoxic conditions have been reported to more effectively inhibit MMP13 expression [26].

Importantly, several studies have highlighted that sEV treatment also increased the expression of the fibrocartilage marker type I collagen (COL1) [13, 17, 34], with a greater increase observed when sEVs were derived from MSCs cultured in 2D rather than 3D environments [13]. Similarly, some authors noted an increase in the expression of the bone-related transcription factor RUNX2 after sEVs treatment [28, 34], and Li et al. (2021) also reported a significant increase in osteocalcin protein expression [34]. Contrary to these findings, two studies reported a reduction in the hypertrophic marker type X collagen (COL10) at both the gene [21] and protein [19] levels following sEV treatment. The expression of fibrous tissue- and bone-related proteins should be closely monitored, and strategies to minimize it explored, as their presence may suggest that sEVs may be promoting endochondral ossification in chondrocytes.

### Chondrocyte inflammatory response

Various studies have reported a reduction in the expression and secretion of inflammatory cytokines such as IL-1β, interleukin-6 (IL-6), and TNF-α following sEVs treatment [21, 22, 32, 34, 38, 40, 41]. One study also noted an increase in the secretion of the anti-inflammatory cytokine interleukin-10 (IL-10) [41]. Palamà et al. (2020) described a reduction in interleukin-1α (IL-1α)-induced production of IL-6, interleukin-8 (IL-8), and cyclooxygenase-2 (COX-2) after 16 h when chondrocytes were co-treated or pre-treated with MSC-derived EVs. However, these protective effects were no longer detectable at 48 h [12]. Other studies reported inhibition of IL-6 and TNF-α secretion after 24 h of sEVs treatment [38, 40, 41], but not beyond. This may suggest that the anti-inflammatory effects of sEVs on chondrocytes may be transient or diminish over time. Further research is needed to determine the optimal dosage of sEVs to achieve sustained anti-inflammatory effects.

### sEVs internalization: chondrocyte cultures vs. cartilage tissue

Many studies have reported that MSC-derived sEVs are efficiently internalized by chondrocyte cultures [13, 19, 21, 22, 25, 32, 33, 36, 37, 40, 42–44], regardless of MSCs preconditioning [12, 22, 25, 32], as fast as 1–6 h [12, 13]. Palamà et al. (2020) found the internalization efficiency to be 31–39%, increasing to 55–66% in an inflammatory microenvironment [12]. Rong et al. (2021) also found that hypoxia enhanced the uptake of MSC-derived sEVs by chondrocytes [26]. In the joint context, Ragni et al. (2020) observed that MSC-derived sEVs interacted with articular chondrocytes, but showed a preferential affinity for fibroblast-like synoviocytes [15].

Notably, most of the in vitro studies mentioned above employed 2D chondrocyte cultures, an approach that does not accurately reflect the conditions of native cartilage, where the ECM constitutes the majority of the tissue, and the cells represent only about 1–2% [45]. In contrast to the findings previously reported, one study conducted on human articular cartilage explants found no significant changes in DNA, glycosaminoglycan, or collagen content after 14 days of sEVs treatment under inflammatory conditions [16]. The study by Ragni et al. (2020) highlighted the crucial role of the ECM in sEVs-cell interactions, which can vary significantly between 2D and 3D environments [15]. Small EVs have been reported to fully penetrate chondrocyte micromasses [14] and to rapidly infiltrate the surface of cartilage explants. However, Colombini et al. (2021) also noted that more sEVs were retained within the ECM than interacted with cells, contrasting with previous observations in micromasses [46]. These findings highlight the importance of using in vitro models that accurately represent cartilage physiology to determine the optimal dosage needed for meaningful clinical impact. According to Mortati et al. (2020), sEVs form a concentration gradient, which makes it challenging for chondrocytes located far from the tissue surface to reach saturation [14]. It is also worth noting that current imaging technologies struggle to differentiate between surface adhesion and actual internalization of sEVs by target cells, an essential distinction for predicting therapeutic efficacy [15].

## Concluding remarks

In summary, growing evidence demonstrates that MSC-derived sEVs exhibit chondroprotective effects, but their efficacy needs to be further investigated in in vitro models that better reflect cartilage pathophysiology. While 2D culture experiments are useful for initial assessments, due to their simplicity and low cost, it is essential to confirm sEVs effects on chondrocytes using in vitro models that closely replicate in vivo conditions. Parameters such as MSC source, culture conditions, and sEVs dosage must be optimized to achieve consistent pro-anabolic and anti-inflammatory effects on chondrocytes. Additionally, standardized sEV isolation methods that ensure consistent yield and high purity should be established.

### In vivo outcomes in Osteoarthritis models

The potential of MSC-derived sEVs for OA treatment has been explored in numerous animal models of cartilage degeneration. This systematic review includes 28 in vivo studies, all of which utilized knee OA models (Table 3). Unsurprisingly, the most commonly represented species was the rat (18 studies), followed by mice (8 studies) and rabbits (2 studies). Collectively, these studies involved a total of 235 joints treated with MSC-derived sEVs and 204 joints treated with the vehicle employed (usually PBS) or scaffold alone. Three studies compared the efficacy of MSCs with their derived sEVs, involving 20 joints treated with MSCs and 20 joints treated with MSC-derived sEVs. Importantly, despite the higher prevalence of knee OA in women, of the 25 studies that specified the sex of the animals, 23 used males, while only 2 used females. Additionally, the vast majority of the disease models were conducted in young rats and mice (8–12 weeks old). Most studies employed surgical OA models, including destabilization of the medial meniscus (DMM; 14 studies), osteochondral defect (6 studies), and groove surgery (1 study). Other authors used monoiodoacetate (MIA; 5 studies) or collagenase (2 studies) injections to induce OA.

**Table 3:** Summary of knee OA models characteristics

Ref.	Producing cells	*Animal model*			Disease model	Administration route	Treatment	Outcomes
		Strain/Sp	Sex	Age				
[25]	SMSCs (human)	C57BL/6 mouse	Male	10 weeks	DMM (ACLT + medial meniscectomy)	Intraarticular	10^9^ EVs/10 µl **Twice a week/6 weeks** **(3 days after surgery)** No further follow-up	Reduced OARSI score Increased ACAN, COL2; reduced ADAMTS5 [IF]
[17]^†^	ADSCs (human)	C57BL/6 mouse	Male	8 weeks	Collagenase-induced OA	Intraarticular	10^9^ CMD4 EVs/8 µL **8 days after CIOA** Follow-up: 20 days	Reduced OARSI score* Reduced macroscopic chondropathy score
[54]	BMSCs (mouse)	C57BL/6 mouse	Male	8 weeks	DMM (ACLT)	Intraarticular	10^8^ EVs/10 µl **Once a week/4 weeks** **(2 weeks after surgery)** No further follow-up	Reduced OARSI score Reduced osteophyte score Reduced MMP13 [IHQ]
[31]^†^	ADSCs (human)	C57BL/6 mouse	Male	10 weeks	DMM (Medial MTL transection)	Intraarticular	10^8^ EVs/6 µl PBS **Once a week/6 weeks** **(5 weeks after surgery)** No further follow-up	Reduced OARSI Score Reduced MMP13 [IHQ]
[32]	ADSCs (mouse)	C57BL/6 mouse	Male	-	DMM (ACLT)	Intraarticular	10^9^ EVs/mL **Twice a week/4 weeks** No further follow-up	Reduced Mankin score* No difference in ACAN; increased COL2 [IHQ]
[55]	UCMSCs (human)	C57BL/6 mouse	Male	12 weeks	DMM (Medial MTL transection)	Intraarticular	10^9^ EVs/5 µL PBS **0**,** 2 and 4 weeks after surgery** Follow-up: 4 weeks	Reduced OARSI/Mankin Score More cartilage in tibial plateau Reduced MMP13, ADAMTS5 [IF]
[58]	UCMSCs (human)	C57BL/6 mouse	Male	8 weeks	DMM (ACLT + medial meniscectomy) Exercise 1 h/day	Intraarticular	10^9^ EVs/10 µL PBS **Twice a week/6 weeks** No further follow-up	Reduced OARSI score Reduced osteophyte formation Increased COL2, ACAN; reduced ADAMTS5/MMP13 [IF] Reduced IL-1β; no changes in TNF-α [ELISA]
[51]	ADSCs (human)	NOD mouse	-	-	Osteochondral defect	Intraarticular	2 µl of EVs (320X) **1 week after surgery** Follow-up: 4 weeks	Increased cartilage repair score Increased proteoglycans; Increased COL2; reduced COL1 [IF]
[56]	UCMSCs (human)	SD Rat	Male	8 weeks	DMM (ACLT)	Intraarticular	200 µl EVs (30 µg) **Once a week/4 weeks** **(4 weeks after surgery)** Follow-up: 1 week	Reduced OARSI score* Increased BVF Reduced MMP13, ADAMTS5 [IHQ]
[37]	UCMSCs (human)	SD Rat	Male	8 weeks	Osteochondral defect	Implantation into cartilage defect	Gel with 10^9^ EVs/mL **(during surgery)** Follow-up: 12 weeks	Increased ICRS histological score* and macroscopic score
[49]	ADSCs (human)	SD Rat	Male	10 weeks	Monoiodoacetate	Intraarticular	CD10High sEVs (secreted by 10^6^ cells)/50 µl EuroCollins **4 days after MIA** Follow-up: 4 days	Reduced histological score* Increased PRG4 superficial expression [IF]
[57]	UCMSCs (human)	SD Rat	Male	6–8 weeks	DMM (ACLT)	Intraarticular	80 µg/mL **Once a week/4–8 weeks** No further follow-up	Reduced Mankin score Reduced macroscopic chondropathy score Reduced MMP13 [IHQ]
[29]	BMSCs (rat)	SD Rat	Male	10 weeks	Osteochondral defect	Implantation into cartilage defect	Gel w/125 µg/kg (sEVs/BW) (100 µL) **(during surgery)** Follow-up: 4–8 weeks	Increased cartilage repair* Increased ICRS macroscopic score No changes in MMP13/COL2 [IHQ]
[50]	SFMSCs (rat)	SD Rat	Male	10 weeks	DMM (ACLT + transverse ligament transection)	Intraarticular	50 µg EVs/100 µL PBS **Once a week/10 weeks** **(8 weeks after surgery)** No further follow-up	Reduced OARSI score Increased COL2; reduced MMP13 [IHQ] Reduced serum inflammatory cytokines [ELISA]
[35]	UCMSCs (human)	SD Rat	-	8 weeks	Monoiodoacetate	Intraarticular	10^8^ EVs/10 µL PBS **Once a week/4 weeks** **(1 week after MIA)** No further follow-up	Reduced Mankin score* No changes in articular space width No changes in subchondral bone (BVF)
[41]	SMSCs (human)	SD Rat	Male	10 weeks	DMM (ACLT + MCL transection)	Intraarticular	3 × 10^9^ EVs/30 µL PBS **Once a week/3 weeks** **(1 week after surgery)** Follow-up: 1 week	Reduced Mankin score* Reduced inflammatory cytokines [ELISA] Reduced apoptosis
[38]	BMSCs (human)	SD Rat	Male	10 weeks	Collagenase-induced OA	Intraarticular	400 µg EVs/mL **1 week after CIOA** Follow-up: 6 weeks	Reduced OARSI/Mankin score*
[26]	BMSCs (rat)	SD Rat	Female	7–8 weeks	DMM (ACLT + medial meniscectomy)	Intraarticular	200 µg EVs/200 µL PBS **4 weeks after surgery** Follow-up: 4 weeks	Reduced OARSI score Reduced osteophyte score Increased COL2 [IHQ/WB]
[42]	UCMSCs (human)	SD Rat	Male	8 weeks	DMM (ACLT)	Intraarticular	30 µg EVs/200 µL PBS **Once a week/4 weeks** **(1 week after surgery)** Follow-up: 4 weeks	Reduced OARSI score* Increased COL2 [IHQ]
[53]	SMSCs (human)	SD Rat	Male	12 weeks	DMM (ACLT + medial meniscectomy)	Intraarticular	2 × 10^10^ EVs/200 µl in hydrogel **Every 4 weeks (x6)** No further follow-up	Regenerated tissue with disorganized arrangement and poor in proteoglycans
[31]^†^	ADSCs (human)	SD Rat	Male	8 weeks	Monoiodoacetate	Intraarticular	10^8^ EVs/30 µl PBS **Subacute**: **Once a week/21 days** **(1 week after surgery)** **Chronic**: **Twice a week/40 days** **(2 weeks after surgery)** No further follow-up	Reduced Modified Mankin score (modest reduction in the chronic model)
[43]	ADSCs (rat)	SD Rat	Male	8 weeks	Osteochondral defect	Implantation into cartilage defect	7.5 × 10^9^ EVs/10 µl gel **(during surgery)** Follow-up: 4–8 weeks	Increased ICRS score* Increased collagen organization Reduced apoptosis Increased BVF and BMD
[27]	BMSCs (human)	SD Rat	Female	4 weeks	DMM (ACLT + medial meniscectomy)	Intraarticular	50–100 µg EVs/100 µL PBS **4 weeks after surgery** Follow-up: 4 weeks	Reduced Modified OARSI score* Increased COL2 [IHQ]
[59]	BMSCs (rabbit)	Wistar Rat	Male	8 weeks	Monoiodoacetate	Intraarticular	50 µg EVs/50 µl PBS **Once a week/4 weeks** **(3 weeks after MIA)** Follow-up: 8 weeks	Increased stride and step length, reduced toe-out angle and gait irregularity Reduced Mankin score* Reduced radiological score Increased COL2 [IHQ]
[47]	BMSCs (rabbit)	Wistar Rat	Male	8 weeks	Monoiodoacetate	Intraarticular	100 µg EVs/50 µl PBS **Once a week/4 weeks** **(3 weeks after surgery)** Follow-up: 12 weeks	Reduced irregularity in gait Reduced histological score* (2D only) Reduced radiological score (2D only)
[52]	BMSCs (human)	Wistar-Han Rat	Male	24 weeks	Groove surgery + metabolic dysregulation (Metabolic OA)	Intraarticular	7.77 × 10^7^ EVs/25 µl PBS **Every 5 days/20 days (x5)** **(8 days after surgery)** Follow-up: 8 weeks	No changes in pain No changes in histology No changes in osteophytes/ subchondral bone Reduced systemic inflammation [ELISA]
[48]	UCMSCs (human)	New Zealand Rabbit	-	-	Osteochondral defect	Intraarticular (EVs) and implantation into cartilage defect (S)	5 µg EVs/200 µl PBS **Once a week/5 weeks** **(3 weeks after surgery)** Follow-up: 2–5 months	Increased histomorphology score* (with fibrillation and other alterations) ↑ ICRS macroscopical score No changes in WORMS Increased BVF (in combination with scaffold)
[44]	BMSCs (human)	New Zealand Rabbit	Male	26 weeks	Osteochondral defect	Intraarticular	3–15 × 10^9^ EVs/300 µl **Once a week/4 weeks** **(1 week after surgery)** No further follow-up	Increased ICRS score* Unsmooth surface Safranin O staining intensity not recovered

DMM: destabilization of the medial meniscus; ATCL: anterior cruciate ligament transection; MTL: meniscotibial ligament; MCL: medial collateral ligament; CIOA: collagenase-induced OA; MIA: monoiodoacetate; BW: body weight; IF: immunofluorescence; IHQ: immunohistochemistry; WB: Western blot; Whole-Organ Magnetic Resonance Imaging Score (WORMS); *: only one articular surface is shown in histology sections; ^†^: competing interests declared by the authors

The sEVs employed in these studies were derived from various MSC sources, with bone marrow being the most common (9 studies; 4 using human BMSCs, 2 using rabbit BMSCs, 2 using rat BMSCs, and 1 using mouse BMSCs), followed by umbilical cord (8 studies, all using human UCMSCs), adipose tissue (7 studies; 5 using human ADSCs, 1 using mouse ADSCs, and 1 using rat ADSCs), synovium (3 studies, all using human SMSCs) and synovial fluid (1 study, using rat SFMSCs). Almost all studies (24) administered sEVs via intra-articular injection targeting the knee joint, while other 3 studies used sEVs-loaded scaffolds for implantation into the cartilage defect, and one study combined scaffold implantation into the cartilage defect with intra-articular sEVs injection. For intra-articular injected sEVs, the dosage varied from 10^8^ to 10^10^ particles and 5 to 200 µg of protein per joint, across a broad range of concentrations (Fig. 2C). It is important to note that in the only two studies reporting yields in terms of both particles per mL and µg of protein per mL [17, 42], the purity of sEVs was relatively low (10^6^-10^7^ particles/µg) according to the scale established by Webber et al. [18].

### Cartilage structure and integrity

Most studies reported improved histological scores and increased expression of cartilage matrix proteins following sEVs treatment. However, it should be noted that 17 studies only showed histological sections of one articular surface, which limits the ability to comprehensively assess cartilage changes across the joint. The weighted mean difference (WMD) in OARSI scores between OA and sEVs treatment was 2.1 ± 0.9 (*n* = 77 joints, data from 11 studies). When considering only those studies with histological sections that showed both articular surfaces, the WMD was 1.7 ± 0.5 (*n* = 50 joints, data from 6 studies). Regarding Mankin scores, the WMD was 4.2 ± 2.0 (*n* = 49 joints, data from 8 studies), which is comparable to the OARSI score WMD when transformed to a 0–6 scale (1.7 ± 0.8). Notably, only 2 of these 8 studies included histological sections showing both articular surfaces. Four studies reported both OARSI and Mankin scores and were included in both analyses.

Mean differences in OARSI scores ranged from 0.8 (in 8-week-old mice) to 4.2 (in 8-week-old rats), with both the lowest and highest values observed in surgical DMM models. For Mankin scores, mean differences ranged from 1.8 (in 8-week-old rats with MIA-induced OA) to 7.5 (in 8-week-old rats subjected to DMM surgery). It is challenging to attribute these differences to the dose of sEVs administered, as these were reported in varying units (particles/mL vs. µg/mL) and likely differed in purity. To add another layer of complexity, the frequency and duration of sEVs administration varied from a single dose to weekly or biweekly doses for up to 8 weeks, with differing follow-up periods.

In addition to these 15 studies, another 10 studies reported improved histological scores in various histopathological grading systems [29, 31, 37, 43, 44, 47–51]. On the contrary, one study employing 24-week old rats with metabolic dysregulation as a model of metabolic mild OA reported no histopathological differences between sEVs- and PBS-treated rats [52]. Another study reported a significant reduction in the modified Mankin score (0–11) in a subacute OA model (mean difference of 8.8), while the change was much more modest in a chronic OA model (mean difference of 2.4). In this study, sEVs treatment led to a significant improvement compared to hyaluronic acid in the subacute model, but not in the chronic model [31].

Many studies reported an increase in the expression of cartilage matrix proteins and proteoglycans, primarily COL2 and ACAN, as well as a decrease in matrix-degrading enzymes, mainly MMP13 and ADAMTS5, as measured by staining and immunostaining techniques. One study reported that sEVs treatment increased the COL2/COL1 ratio [51], while another observed an increase in lubricin (PRG4) expression in superficial cartilage cells [49]. Conversely, a few studies showed no changes in COL2 [29], ACAN [43], or MMP13 [29] expression following sEVs treatment. One study noted that despite an increase in the histomorphology score, the cartilage of sEVs-treated animals exhibited fibrillation, increased cell content compared to sham, and a lack of organized chondrocyte columns [48]. Other studies reported that regenerated tissue in sEVs-treated animals displayed a disorganized arrangement [53], with no recovery of proteoglycans [44, 53] or an unsmooth surface [44]. Another study indicated that the administration of MSC-derived sEVs resulted in hypertrophic cartilage, with levels of COL10 comparable to those in the untreated control [47].

Interestingly, several studies showed that preconditioning of MSCs with inflammatory *stimuli* [25], antioxidants [32], hypoxia [26, 27], or electromagnetic field [54] before harvesting sEVs enhanced their beneficial effects on cartilage structure and integrity. Genetic engineering approaches, such as the inclusion of miRNAs and cartilage-targeting molecules in sEVs, have also been reported to increase efficacy [35]. In addition, the use of scaffolds can also improve sEVs performance. The study by Jiang et al. (2021) demonstrated better cartilage repair when combining weekly sEVs injections with implantation of an acellular cartilage matrix compared to each treatment alone [48]. Similarly, sEVs-loaded hydrogels achieved better ICRS scores than hydrogels alone in two independent studies [37, 43]. Of note, a few studies showed that sEVs treatment was equal [55, 56] or even superior [42] to MSC treatment, supporting the notion that paracrine signalling mediates the beneficial effects of MSCs on OA [2]. Moreover, sEVs have been reported to yield better outcomes than large EVs [43] and platelet-rich plasma [57]. One study demonstrated that MSC-derived sEVs preferentially target superficial chondrocytes and fibroblast-like synoviocytes, which are key cells in the joint environment [55].

### Subchondral bone remodelling

Regarding OA-related subchondral bone changes, one study found an increased bone volume fraction (BVF) in both MSC- and sEVs-treated rats [56], while other authors reported no changes in BVF after a similar sEVs administration schedule (once a week for four weeks) [35]. Again, it is not possible to determine if the sEV doses were comparable because they were reported in different units (30 µg vs. 10^8^ particles) and purity was not specified. Another study with a similar administration schedule failed to find an increase in BVF due to sEVs treatment alone, but demonstrated that sEVs treatment increased BVF in animals implanted with an acellular cartilage matrix scaffold into the osteochondral defect. However, the improvement compared to animals treated with the scaffold alone was moderate [48]. In another study in which sEVs were loaded into a hydrogel that was implanted in the osteochondral defect during surgery, improvements in both BVF and bone mineral density (BMD) were noted as soon as 4 weeks after treatment [43]. Various studies found that sEVs treatment reduced osteophyte formation [26, 54, 58], and this effect was enhanced when MSCs were preconditioned in hypoxia [26] or subjected to electromagnetic field stimulation [54]. In contrast, sEVs induced no changes in subchondral bone structure or osteophyte formation in the metabolic mild OA model used by Warkmik et al. (2023) [52].

### Macroscopic and functional outcomes

Some studies have reported that sEVs treatment lowered macroscopic chondropathy scores [17, 57] or increased ICRS macroscopic scores [29, 37, 48], especially when sEVs were loaded into scaffolds [37, 48]. Two studies also noted better radiological scores following sEVs treatment, with further enhancement observed when MSCs were co-cultured with chondrocytes [47, 59], while another study found no differences in articular space between PBS- and sEVs-treated rats [35]. Importantly, improvements in gait parameters after sEVs treatment were also observed, including longer stride and step lengths [59] and reduced gait irregularity [47, 59]. Again, no significant differences in pain were noted between sEVs- and PBS-treated rats in the metabolic mild OA model [52].

### Inflammatory microenvironment

Notably, sEVs treatment has been shown to reduce inflammatory cytokine levels both locally [41] and systemically [50, 52] in surgical OA models, including the metabolic mild OA model [52]. Inflammatory cytokines, such as IL-1β and TNF-α, stimulate the production of matrix-degrading enzymes, impair chondrocyte function, and promote osteophyte formation. Therefore, mitigating inflammation is crucial to preventing cartilage degradation. Importantly, one study demonstrated that sEVs treatment had no effect on NLRP3^−/−^ mice in terms of OARSI scores and osteophyte formation [58], suggesting that the therapeutic effects of MSC-derived sEVs are mediated through the NLRP3 inflammasome pathway. Hypothetically, NLRP3 inhibition induced by the cargo of MSC-derived sEVs would reduce the release of inflammatory cytokines and matrix-degrading enzymes, thereby creating a favourable environment for cartilage repair.

## Concluding remarks

Despite the beneficial outcomes reported, several studies suggest that if severe cartilage degradation has already occurred at the treatment onset, or in more complex OA subtypes (e.g., metabolic or chronic OA) [31, 52], the anti-inflammatory and pro-anabolic effects of MSC-derived sEVs may be insufficient to significantly improve cartilage structure and function. In order to determine whether sEVs are a useful therapy for OA, further research needs to be performed in animal models that more closely resemble OA patients, such as aged animals, animals with obesity and/or metabolic conditions, and, especially, including female animals. Additionally, it is crucial to adjust sEVs dosage and comprehensively assess all factors related to OA progression: cartilage structure, intra-articular space, subchondral bone integrity, and, most importantly, joint function and pain. Finally, strategies such as combination with biomaterials or genetic engineering approaches may be useful to increase efficacy.

### Molecular insights into sEVs-Induced cartilage repair

Numerous studies have shown that specific miRNAs and proteins within sEVs can regulate diverse signalling pathways involved in inflammation, proliferation, and differentiation, including the PI3K/AKT/NF-κB pathway [21, 22, 34, 42, 57], the Wnt/β-catenin pathway [40], the Hippo-YAP pathway [33], and the ECM-receptor interaction pathway [42, 56]. Specifically, the miRNA cargo of MSC-derived sEVs is thought to be a significant contributor to their chondroprotective effects, and many different miRNAs have been attributed chondroprotective properties.

The let-7 family of miRNAs are ones of the most abundant miRNAs in sEVs derived from different MSC sources, including bone marrow [23], adipose tissue [31], umbilical cord [48, 56, 57], and synovium [25]. Specifically, let-7a-5p and let-7b-5p were highly expressed in sEVs derived from BMSCs [23], ADSCs [31], and UCMSCs [48, 57]. In addition, let-7i-5p was enriched in BMSC-sEVs [23], while let-7c-5p, let-7e-5p, and let-7f-5p were enriched in UCMSC-sEVs [48, 56]. Importantly, dysregulation of the let-7 family of miRNAs can contribute to articular cartilage degeneration [60]. In SMSC- sEVs, let-7b-5p was found to be responsible of the inhibition of ADAMTS5. Moreover, this miRNA was highly upregulated by LPS pre-treatment, which could be related to the superior chondroprotective effects of these sEVs [25]. Additionally, Chen et al. (2022) suggested a role for let-7e-5p in promoting cartilage repair through IGF1R/ STAT3 signalling [56]. Notably, the expression of this miRNA is decreased in knee osteoarthritis, correlating with elevated apoptosis and reduced autophagy [61].

Several other miRNAs were commonly highly expressed in sEVs derived from MSCs from different sources; for instance, miR-23a-3p, miR-125b-5p, and miR-199a-3p were also enriched in BMSC-, ADSC- and UCMSC-derived sEVs [23, 31, 37, 56], and mir-92a-3p was commonly enriched in ADSC- and UCMSC-derived sEVs [31, 56]. In the study by Hu et al. (2020), miR-23a-3p was the most abundant miRNA in UCMSCs- sEVs and prevented chondrocyte apoptosis by suppressing PTEN [37]. Regarding the other miRNAs, miR-125b-5p is a negative co-regulator of MMP13 via the TRAF6/MAPKs/NF-κB pathway [62], while miR-92a-3p inhibits the expression of ADAMTS-4/5 [63], and miR-199-3p enhances chondrocyte proliferation and inhibits apoptosis via DNA methyltransferase 3 A (DNMT3A) repression [64]. Furthermore, the expression levels of these three miRNAs have previously been reported to decrease in OA [31, 64].

Other miRNAs with relevant functions in the context of cartilage repair may include: miR-26a-5p, which is carried by SMSC-EVs and inhibits PTEN, supressing apoptosis [41]; miR-27b-3p, which targets leukaemia inhibitory factor (LIF) [55]; miR-99b, which decelerates OA progression through the MFG-E8/NF-κB signalling axis [54]; miR-216a-5p, which enhances the viability and proliferation of chondrocytes by activating the JAK2/STAT3 signalling pathway [26]; miR-217, which acts via EZH2/FOXO3 to restrain apoptosis and promote autophagy [30]; miR-223, which increases in sEVs-treated knees and directly targets NLRP3 [35]; and miR-1208, which inhibits METTL3 expression, also reducing NLRP3 activity [58]. Interestingly, the treatment of ADSCs with IL-1β upregulates miRNAs with chondroprotective potential in their derived sEVs [46]. In the same way, sEVs derived from hypoxia preconditioned MSCs were enriched in miRNA-18-3p and miRNA-181c-5p, which can promote chondrocyte proliferation through the JAK/STAT or MAPK signalling pathways [27].

## Conclusions and future directions

The extensive investigation of MSC-derived sEVs in diverse in vitro and in vivo models has provided valuable insights into their chondroprotective effects. While many studies report that sEVs enhance chondrocyte viability, proliferation, and migration while reducing inflammation, catabolism, and apoptosis, the variability in cell sources, sEV dosages, and experimental conditions highlights the need for further standardization. To move closer to potential clinical applications, sEVs isolation methods should be systematically investigated to identify those that provide consistent yields and high purity. For this purpose, yield should be reported in terms of both the number of particles and the quantity of protein per mL of the original culture medium and per producing cell, and cell culture parameters should be reported according to ISEV guidelines [65]. From a clinical practicality perspective, focusing on less invasive MSC sources, such as the umbilical cord, can be advantageous [6].

Regarding in vitro outcomes, the discrepancies found between 2D and 3D models, particularly concerning sEVs internalization and distribution within the cartilage, emphasize the importance of using experimental models that closely mimic in vivo cartilage physiology. Ideally, these models should replicate both the ECM and cellular organization characteristic of articular cartilage. In addition to cartilage explants, 3D chondrocyte cultures embedded in cartilage-mimicking scaffolds, as well as bioprinted cartilage models with zonal architecture, may fulfil these requirements. Furthermore, organ-on-a-chip models integrating cartilage and other joint tissues, such as bone and synovium, may offer valuable insights into sEVs tissue distribution and efficacy, while also allowing for the simulation of biomechanical forces that mimic physiological or pathological joint loading.

Overall, the outcomes of animal models suggest that, while EVs hold promise in promoting cartilage repair and reducing inflammation in OA, there are still limitations in their effectiveness, particularly in severe cartilage degradation and complex OA subtypes. Importantly, despite its relevance, the biodistribution and localisation of sEVs following in vivo administration is often neglected by researchers [6]. Among the studies included in this review, only one investigated the tissue distribution of the injected sEVs, reporting that they preferentially targeted superficial chondrocytes and fibroblast-like synoviocytes [55]. Future research should focus on identifying the target cells of sEVs within the joint and determining the depth to which sEVs can penetrate the cartilage.

Further investigation is also required to optimise sEVs dosage to enhance tissue regeneration and achieve reliable, long-term therapeutic outcomes. In line with these priorities, it is crucial to evaluate the efficacy of sEV treatments in animal models that accurately represent OA patients in terms of age, gender, and comorbidities. Moreover, in vivo studies should report changes in joint function and pain, which are key parameters for assessing treatment performance. A few studies have suggested marginally better outcomes with sEVs compared to other therapies, such as platelet-rich plasma [57] and hyaluronic acid [31], but further research is necessary to confirm these effects. It is also essential that negative results, such as those observed in the metabolic mild OA model [52], are reported, as they are often only published when compared to a strategy that demonstrates improved outcomes [47, 48, 53].

**Fig. 3:**
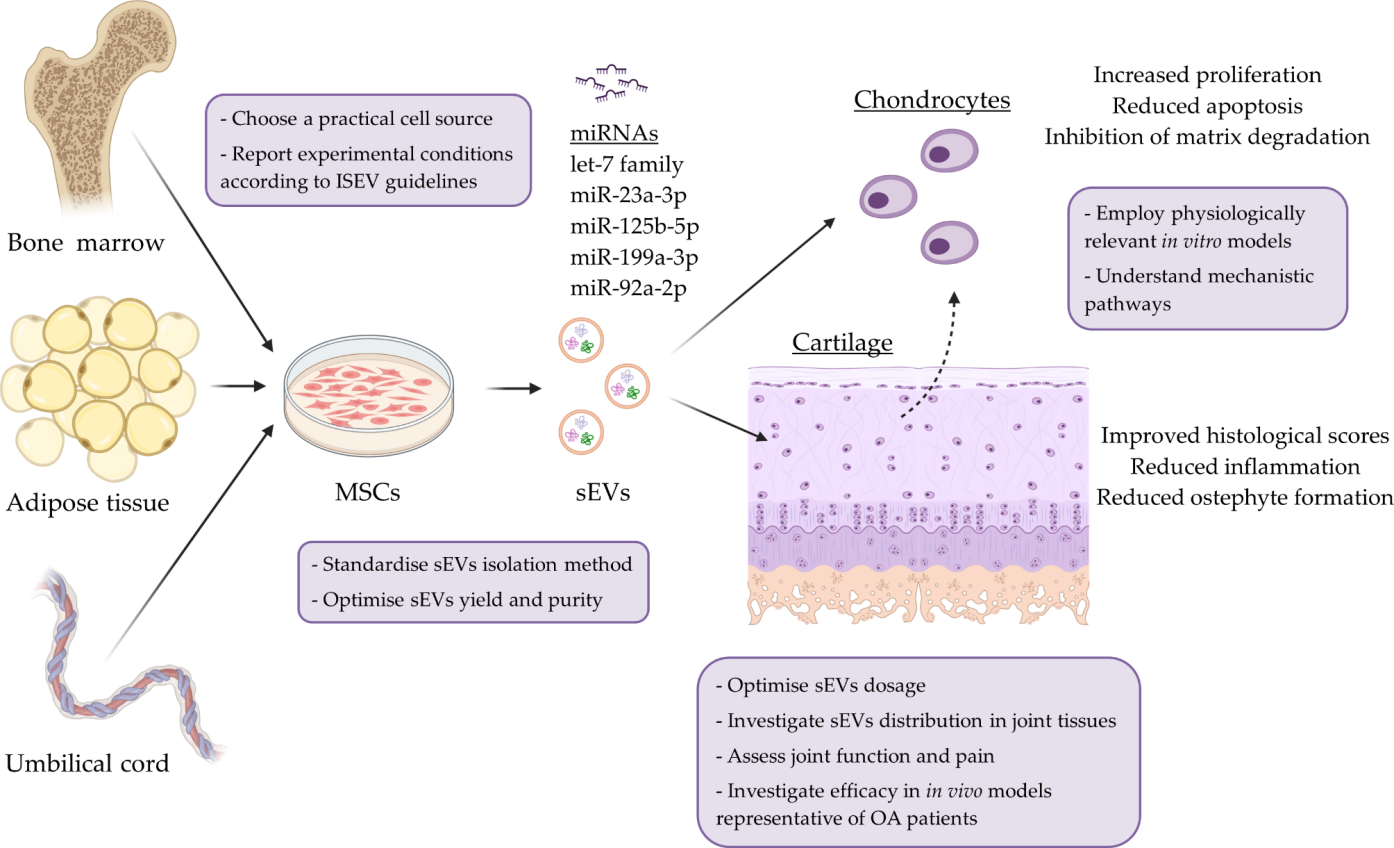
Diagram showing the main effects of MSC-derived sEVs on chondrocytes and cartilage, the most abundant miRNAs across different MSC sources, and recommendations for future research

Given the numerous parameters that still need to be optimized (e.g., sEV obtainment, dosage, and efficacy) and the largely unknown mechanisms of action, clinical translation, despite significant research efforts to date, remains a distant prospect. Nonetheless, by addressing these challenges and adhering to ISEV guidelines [4, 65], as well as the “nanodiamond concept” proposed by Karoichan et al. (2024) (which advocates for a relevant and practical sEVs source, standardised and reproducible methods, intended localization and biodistribution, sustained therapeutic effects, and comprehensive disease management ) [6], and the recommendations outlined above, future research can pave the way toward unlocking the full therapeutic potential of sEVs for cartilage regeneration (Fig. 3).

## Electronic supplementary material

Below is the link to the electronic supplementary material.

Supplementary Material 1

## Data Availability

All data generated or analysed during this study are included in this published article and its supplementary information files.

## **Declarations**

The authors declare that they have not use AI-generated work in this manuscript.

## Abbreviations

OA: Osteoarthritis
MSCs: Mesenchymal stromall cells
EVs: Extracellular vesicles
sEVs: Small EVs
ECM: Extracellular matrix
BMSCs: Bone marrow MSCs
ADSCs: Adipose-tissue derived MSCs
UCMSCs: Umbilical cord MSCs
SMSCs: Synovium-derived MSCs
SFMSCs: Synovial fluid MSCs
DSCs: Dental follicle cells
NPCs: Nucleus pulposus cells
IVDD: Intervertebral disc degeneration
IL: 1β-Interleukin-1β
LPS: Lipopolysaccharide
TGF: β-Tumour growth factor-β
TNF: α-Tumour necrosis factor-α
IFN: α-Interferon-α
ACAN: Aggrecan
COL2: Type II collagen
MMPs: Metalloproteinases
COL1: Type I collagen
COL10: Type X collagen
IL: 6-Interleukin-6
IL: 10-Interleukin-10
IL: 1α-Interleukin-1α
IL: 8-Interleukin-8
COX: 2-Cyclooxygenase-2
DMM: Destabilization of the medial meniscus
MIA: Monoiodoacetate
WMD: Weighted mean difference
BVF: Bone volume fraction
BMD: Bone mineral density
